# Correction: Chen et al. Dental Pulp Stem Cell-Derived Factors Alleviate Subarachnoid Hemorrhage-Induced Neuroinflammation and Ischemic Neurological Deficits. *Int. J. Mol. Sci.* 2019, *20*, 3747

**DOI:** 10.3390/ijms27219734

**Published:** 2026-10-30

**Authors:** Te-Fu Chen, Kuo-We Chen, Yueh Chien, Ying-Hsiu Lai, Sung-Tsang Hsieh, Hsin-Yi Ma, Kou-Chung Wang, Chia-Yang Shiau

**Affiliations:** 1 Department of Surgery, Division of Neurosurgery, National Taiwan University Hospital, Taipei 100, Taiwan; tefu.chen@caire.com.tw (T.-F.C.);; 2 Graduate Institute of Medical Sciences, National Defense Medical Center, Taipei 114, Taiwan; 3 Department of Neurosurgery, Tri-Service General Hospital, Taipei 115, Taiwan; 4 Non-Invasive Cancer Therapy Research Institute-Taiwan, Taipei 104, Taiwan; 5 Institute of Pharmacology, School of Medicine, National Yang-Ming University, Taipei 112, Taiwan; 6 Department of Medical Research, Taipei Veterans General Hospital, Taipei 112, Taiwan; 7 Department of Anatomy and Cell Biology, College of Medicine, National Taiwan University, Taipei 102, Taiwan

## Error in Figure

In the original publication [1], there was a mistake in Figure 2 as published. During the preparation of Figure 2 (aSAH panel), a vascular image was inadvertently reused from a previous experimental series. The corrected Figure 2 has been replaced with a comparable vascular image obtained from a different experimental animal under identical SAH conditions. The corrected Figure 2 appears below.

The authors state that the scientific conclusions are unaffected. This correction was approved by the Academic Editor. The original publication has also been updated.

## Figures and Tables

**Figure 2:**
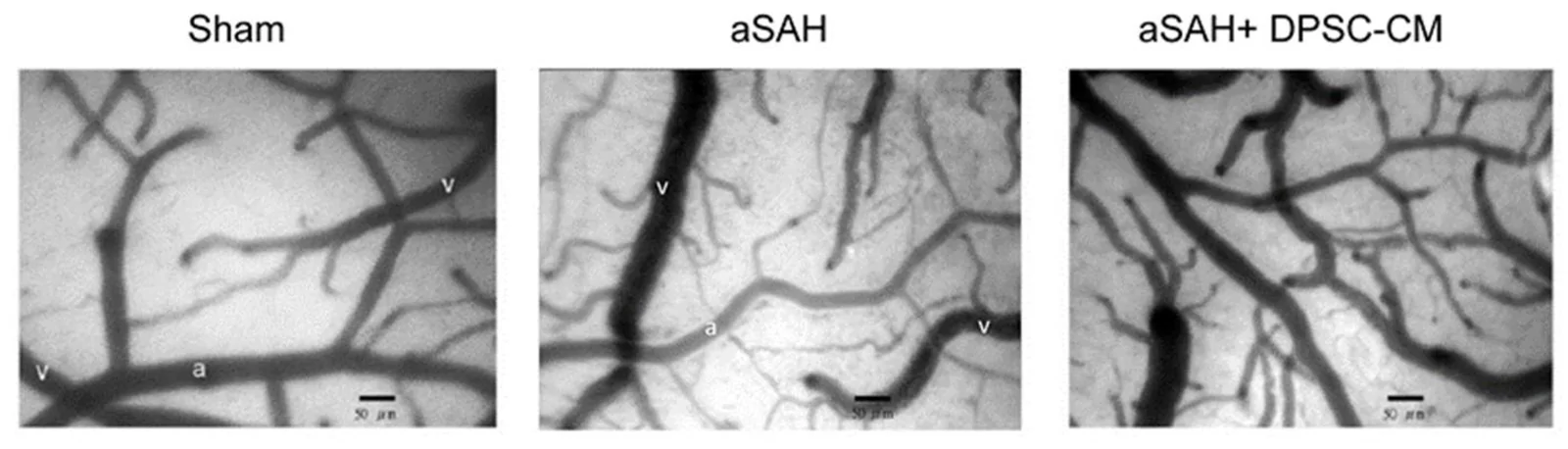
Amelioration of experimental aSAH by foramen magnum injection of DPSC-CM. Rat aSAH model was induced by the injection of fresh autologous blood into the cisterna magna. Induction of aSAH led to a diffuse vasoconstriction, predominantly in secondary and terminal cortical arterioles (middle). Administration of DPSC-CM via foramen magnum at the onset of aSAH induction effectively ameliorated the diffuse vasoconstriction (right). aSAH: aneurysmal subarachnoid hemorrhage. DPSC-CM: DPSC-derived conditioned media. “a”: artery. “v”: vein.
